# Maternal psychological responses during pregnancy after ultrasonographic detection of structural fetal anomalies: A prospective longitudinal observational study

**DOI:** 10.1371/journal.pone.0174412

**Published:** 2017-03-28

**Authors:** Anne Kaasen, Anne Helbig, Ulrik F. Malt, Tormod Næs, Hans Skari, Guttorm Haugen

**Affiliations:** 1 Department of Nursing and Health Promotion, Oslo and Akershus University College of Applied Sciences, Oslo, Norway; 2 Department of Obstetrics, Oslo University Hospital, Oslo, Norway; 3 Department of Research and Education, Division of Clinical Neuroscience, Oslo University Hospital, Oslo, Norway; 4 Institute of Clinical Medicine, University of Oslo, Oslo, Norway; 5 Nofima Food Research, Ås, Norway; 6 Department of Food Science, Spectroscopy and Chemometrics, University of Copenhagen, Copenhagen, Denmark; 7 Department of Gastrointestinal- and Pediatric Surgery, Oslo University Hospital, Oslo, Norway; National Cancer Center, JAPAN

## Abstract

In this longitudinal prospective observational study performed at a tertiary perinatal referral centre, we aimed to assess maternal distress in pregnancy in women with ultrasound findings of fetal anomaly and compare this with distress in pregnant women with normal ultrasound findings. Pregnant women with a structural fetal anomaly (n = 48) and normal ultrasound (n = 105) were included. We administered self-report questionnaires (General Health Questionnaire-28, Impact of Event Scale-22 [IES], and Edinburgh Postnatal Depression Scale) a few days following ultrasound detection of a fetal anomaly or a normal ultrasound (T1), 3 weeks post-ultrasound (T2), and at 30 (T3) and 36 weeks gestation (T4). Social dysfunction, health perception, and psychological distress (intrusion, avoidance, arousal, anxiety, and depression) were the main outcome measures. The median gestational age at T1 was 20 and 19 weeks in the group with and without fetal anomaly, respectively. In the fetal anomaly group, all psychological distress scores were highest at T1. In the group with a normal scan, distress scores were stable throughout pregnancy. At all assessments, the fetal anomaly group scored significantly higher (especially on depression-related questions) compared to the normal scan group, except on the IES Intrusion and Arousal subscales at T4, although with large individual differences. In conclusion, women with a known fetal anomaly initially had high stress scores, which gradually decreased, resembling those in women with a normal pregnancy. Psychological stress levels were stable and low during the latter half of gestation in women with a normal pregnancy.

## Introduction

Ultrasonographic detection of a fetal anomaly in pregnancy causes parental psychological stress. [1, 2] To improve interventions to reduce distress, we need longitudinal information on different aspects of distress (e.g., emotional, cognitive, and behavioural data). In a study from Thailand among women carrying a fetus with an anomaly, only levels of anxiety were assessed. [3] High levels of anxiety, as measured by Spielberger’s State Anxiety Inventory, were present in the second trimester, then transiently decreased and finally increased in the last month before term (37 weeks). Nes et al. [4] conducted a population-based epidemiological study but restricted their report to Down syndrome and cleft lip and/or palate. Questionnaire data (a short version of Hopkins Symptoms Checklist 25-item) collected at recruitment (17−18 weeks) and week 30 showed that women carrying an abnormal fetus had moderate levels of distress that was stable (Down syndrome) or decreasing (cleft lip and/or palate) in the third trimester compared to women carrying a normal fetus.

Cross-sectional studies have found that anxiety and other maternal stress levels are closely correlated to the diagnostic uncertainty and the severity of the anomaly. [5] However, knowledge about the relationship between aspects of the fetal anomaly and the type and level of distress during the course of pregnancy is lacking. This information is needed to design and conduct optimal interventions that will reduce the distress associated with fetal anomaly.

Previously, our group reported the association of maternal psychological distress at the time of detection of a fetal malformation and the severity of the anomaly, diagnostic and prognostic ambiguity, and gestational age. [2] The aim of the present study was to assess the level of emotional, behavioural, and cognitive distress in a subgroup of patients with detected fetal anomaly and to compare this with the level of distress in women with a fetus without anomaly, at four different points in time during pregnancy. We hypothesised that emotional, behavioural, and cognitive responses would be highest at the time of detection and then decrease as pregnancy progressed. We also wanted to study the relationship between distress responses over time and the severity of the anomaly, diagnostic and prognostic ambiguity, and gestational age.

## Materials and methods

### Participants

Pregnant women receiving obstetric care at our tertiary perinatal care centre were consecutively recruited into one of two groups: the group with a fetal anomaly detected by ultrasound (study group) or the group with normal ultrasound findings (comparison group). We used convenience sampling, depending on workload. The sampling was similar for both groups. Women were excluded if they were not fluent in Norwegian, were under 18 years, had a clinical psychiatric diagnosis (e.g., psychosis, severe bipolar disorder, or drug abuse), or expected a multiple birth. Written, informed consent was obtained before participation.

Women in the study group were recruited from May 2006 to August 2009, among pregnant women referred to a tertiary referral centre following the identification of a suspected structural fetal anomaly during obstetric ultrasound examination. Of 180 [2] women with a fetal anomaly who were eligible for study, 87 terminated the pregnancy and 34 were identified too late (i.e. > 27 gestational weeks) to complete the study assessments. Thus, we included 59 women with confirmed fetal structural anomaly.

The comparison group were recruited from April 2007 to February 2009 among women with normal findings on routine ultrasound scan and no history of fetal anomalies or severe obstetric complications. This scan was performed by midwifes trained in fetal sonography.

Finally 111 women met the criteria for entry into the comparison group.

The study assessments consisted of psychometric distress measurements, followed by a consultation and ultrasound examination by a fetal medicine specialist. Four assessments were carried out at different time points: The first assessment (T1) was completed within a few days following detection of a fetal anomaly or a normal finding on ultrasound examination. The second assessment (T2) was performed 2–3 weeks after T1. The third (T3) and fourth (T4) assessments were done at 30 and 36 weeks gestation, respectively.

We recorded medical and obstetric history, socio-demographic variables, gestational age, and the tentative ultrasound diagnosis. Any changes in the fetal diagnosis or prognosis between assessments were recorded.

### Ultrasound examination and counselling

Fetal medicine specialists performed the ultrasound examinations. After the ultrasound examination, the fetal medicine specialist counselled all women in the study group, and specialists in neonatology, pediatric surgery, pediatric cardiology, neurosurgery, or medical genetics were additionally consulted, as needed. The women with a fetal anomaly received close maternal and fetal follow up throughout the pregnancy.

### Fetal anomaly

Fetal diagnoses at T1 were classified, according to Kaasen et al. [2] with respect to severity and diagnostic or prognostic ambiguity at the time of recruitment. A prognosis was defined as ambiguous if: a) the anomaly had significant inherent prognostic variation, or b) a definite diagnosis depended on the results of further investigation (e.g., an invasive test). If further investigations were assumed to be important for the diagnosis or prognosis, the severity was categorised as ‘not classified; anomaly awaiting clarification’. Three of the authors performed the classification, with strong inter-rater agreement (κ = 0.86). [2]

A fetal anomaly was categorised as:

1. Lethal or serious with no available treatment, with or without prognostic ambiguity (e.g., acrania, skeletal dysplasia with small thorax, holoprosencephaly)
2. Serious with available treatment, with prognostic ambiguity (e.g., myelomeningocele with hydrocephalus, hypoplastic left heart syndrome)
3. Mild to moderate severity with available treatment, often with good result, but with prognostic ambiguity (e.g., bilateral clubfoot or cleft lip with no other markers, condition known to be associated with syndromes not apparent prenatally)
4. Mild to moderate severity with available treatment, often with good result, without prognostic ambiguity (e.g., gastroschisis, unilateral clubfoot)
5. Severity not classified; awaiting clarification. Prognosis highly dependent on the results of an invasive test (e.g., omphalocele, bilateral clubfoot with chromosomal soft markers), or a reliable diagnosis was not available at inclusion because of an incomplete ultrasound examination (e.g., maternal obesity)

‘Not classified, anomaly awaiting clarification’ corresponds to an inconclusive ultrasound examination at the referral center. Fourteen of the 18 women with this classification had an invasive fetal diagnostic test before T2, and they all received an answer before T2. Four women decided not to have an invasive test. In two of these women, the fetal prognosis became worse, in six the prognosis improved, and in 10, the prognosis was stable.

Throughout pregnancy, any changes in fetal diagnosis and/or prognosis were recorded as ‘improved’, ‘stable’, or ‘worse’. “Improved’ signified that the fetal anomaly was determined to have less influence on the child’s future health compared with a previous assessment (e.g., following receipt of a normal karyotype). The diagnosis or prognosis was considered ‘worse’ following the finding of an abnormal karyotype or following additional observations showing worsening of the fetal condition on repeated ultrasound examinations.

### Psychometric questionnaires

We used three psychometric questionnaires, the General Health Questionnaire (GHQ), [6] the Impact of Event Scale (IES), [7] and Edinburgh Postnatal Depression Scale (EPDS). [8]

The GHQ [6] is a 28-item scale consisting of four seven-item subscales measuring social dysfunction, health perception (somatic symptoms) and anxiety and severe depressive symptoms (the two last subscales measuring psychological distress) during the preceding two weeks. We used Likert scoring (summing the individual item scores graded from 0–3, which gave possible total scores ranging from 0–84) to compare distress levels within and between groups. We also calculated GHQ ‘case’ scores by dichotomising the responses (e.g., less/much less vs same/better) for each of the 28 items—the sum GHQ case score provided an estimate of the prevalence of clinically significant psychological distress (defined as a sum case score ≥6 out of a possible score of 28). In particular, the GHQ items 24 (‘life is not worth living’), 25 (‘considering ending my life’), 27 (‘wished I was dead’), and 28 (‘thinking about ending my life’) were used to assess suicidal ideation (based on a Likert score of 2–3 on any item). [6] Other research groups have previously used the GHQ for assessment of stress in pregnancy. [9, 10]

IES is a questionnaire measuring emotional and behavioral responses to stressful events during the previous week. The original IES scale had 15 items measuring emotional and behavioral responses to a traumatic event, i.e. intrusion (seven items) and avoidance (eight items). [7] Intrusion is characterised by unbidden thoughts and images, troubled dreams, strong waves of feelings, and repetitive behaviour (related to the experience of knowing about the fetal condition, in the case of the study group). Avoidance is characterised by ideational constriction related to the fetal condition, denial of the consequences of the anomaly, blunted sensations, behavioural inhibition, and awareness of emotional numbness. The IES-22 version used in this study includes 6 additional items measuring arousal and 1 additional item measuring intrusion as published by Weiss & Marmar 1997. [11] Arousal measures distress-associated, psycho-physiological activation and is characterized by anger and irritability, a heightened startle response, concentration difficulties, and hypervigilance. However, in contrast to the Weiss and Marmar IES-22-R version, we did not reduce the point level for each item to 0–4 [12], but applied the original 0 -5-point endorsement levels for each item. Thus, the IES applied in our study yields a score range of 0–40 for intrusion (Cronbach’s Alpha 0.807) and avoidance (Cronbach’s Alpha 0.812), and 0–30 for arousal (Cronbach’s Alpha 0.735). Intrusion and avoidance scores < 9 is considered to be within the normal ranges, while 9–19 is considered a sub-threshold response. ≥20 indicates intrusion and avoidance responses of definite clinical importance. Previous studies have used the IES-22-R for assessing stress in pregnancy. [13, 14]

The EPDS [8] consists of ten questions and is a self-report scale designed to detect postnatal depression. Five of the items measure dysphoric mood, two measure anxiety, and one each measure guilt, suicidal ideas, and incidence of ‘not coping’ experienced during the previous week. The EPDS has been validated for use in pregnancy, too. [15] We calculated total EPDS scores (individual items were scored 0–3, giving a possible total score of 30), where an EPDS total score ≥10 was associated with mild depressive symptoms and a score of ≥13 was used to identify moderate or more severe symptoms of depression. [16] The EPDS items 10 (‘The thought of harming myself has occurred to me’), was used to assess suicidal ideation.

The questionnaires were completed at the hospital, and the participants were instructed to complete the questionnaires without discussing the questions with others.

### Statistics

The sample size was calculated according to Skari et al. [17] who reported that GHQ total Likert scores differed by ⅔ SD for parents of a fetus diagnosed at 25–30 weeks of gestational age compared with those diagnosed earlier in pregnancy. Accordingly, each group required 40 patients to obtain the same difference with α = 0.05 and a statistical power of 85%.

Completed questionnaires were scanned using Cardiff TeleForm version 10.1 (Autonomy Corporation plc, Cambridge, England) and stored in Access 97 (Microsoft Corporation, Redmond, WA, USA). SPSS version 22 (IBM, Armonk, NY, USA) was used for statistical analyses.

For descriptive statistics, within and between the two groups of women, we used parametric or non-parametric analyses, as appropriate. Analysis of variance (ANOVA) was performed separately for each of the independent variables to identify predictors of psychosocial distress. Continuous variables were transformed into categorical variables (clinically relevant groups). In a separate analysis, we explored the subgroup of women with scores ≥3 for any of the 22 items of the IES and ≥1 for any of the ten items of the EPDS. With regard to the GHQ, we only investigated suicidal ideation in detail because of a low variation in the GHQ total scores.

### Ethical issues

Written, informed consent was obtained before participation. The Regional Ethics Committee of Southern Norway approved the study December 21^st^ 2005 and May 10^th^ 2016 (Reference number S-05281, 2016/779/REK sør-øst). The Institutional Review Board approved the study.

In accordance with the study protocol, any participant with a case score of ‘1’ on at least one of the four GHQ items addressing suicidal ideation was contacted for clinical evaluation on the same day and if necessary, offered psychiatric assistance.

## Results

The women in the study group (n = 59) had a median gestational age of 20 weeks, 2 days (range 12–26 weeks) at recruitment. The women in the comparison group (n = 111) were recruited at a median of 19 weeks, 3 days (range 12–22 weeks).

In the study group, 48 of 59 women attended all four assessments. The others experienced fetal loss (n = 5), preterm birth prior to T4, i.e., <36 gestational week (n = 3), withdrew from the study (n = 2), or were lost to follow up (n = 1). In the comparison group, 105 women attended all four assessments, whereas three women delivered prematurely (before T4), two did not attend all four assessments, and one woman withdrew from the study.

The groups were similar; however, the study group had significantly lower educational level and a larger variance in gestational age at recruitment than did the comparison group (Table 1).

**Table 1:** Characteristics for women in group with and without fetal anomaly at inclusion (T1).

		Women with all four assessments/visits		
		Fetal anomaly (n = 48) N (%)	No fetal anomaly (n = 105) N (%)	P-value*
**Age**	19–28 years	17 (35)	25 (24)	0.223
	29–33 years	20 (42)	44 (42)	
	34–43 years	11 (23)	36 (34)	
**Education**	< junior college	23 (48)	16 (15)	<0.001
	≥ junior college	25 (52)	88 (85)	
	Missing data	0	1 (1)	
**Previous children**	No previous children	20 (42)	59 (56)	0.135
	Previous children	28 (58)	46 (44)	
**Married or cohabiting**	Yes	46 (96)	105 (100)	n/a
	No	2 (4)	0	
**Gestational age at first assessment**	<18 weeks	11 (23)	16 (15)	0.004
	18–22 weeks	33 (69)	89 (85)**	
	>22 –<27 weeks	4 (8)	0	
**Time from suspicion of fetal anomaly to examination at the referral center**	≤ 2 days	36 (75)	n.a.	
	3–4 days	5 (10)		
	≥ 5 days	7 (15)		
**Change in diagnosis/prognosis from T1 to T2**	Improvement	18 (38)	0	<0.001
	Stable	28 (58)	104 (99)	
	Worsening	2 (4)	1 (1)***	
**Classification of severity (see text)**	1	0	n/a	
	2	6 (13)		
	3	9 (19)		
	4	15 (31)		
	5	18 (38)		

* Chi-Square tests.

** In the group without fetal anomaly one case was moved to the 18–22 weeks group due to statistical purposes. This case was included in the study at week 22 + 1 day of gestation.

*** One anomaly in the comparison group detected at T 3 (a cyst in one thigh).

Compared with the comparison group, the study group had high distress scores on several of the GHQ subscales, anxiety at all four assessments, depression at the three first assessments, social dysfunction at the two first assessments, and disturbed health perception (somatic symptoms) at the second assessment (Table 2).

**Table 2:** Psychometric scores in women with and without a fetal anomaly, assessed at four points in pregnancy.

			Study group N = 48 (fetal anomaly)			Comparison group N = 105 (healthy)			P-value*
			Median (min-max)	Mean (SD)	N	Median (min-max)	Mean (SD)	N	
**Time 1**	**GHQ**	Sum Likert	25.5 (10–59)	26.6 (10.7)	48	19.0 (8–59)	19.9 (8.2)	105	<0.001
		Health perception	6.0 (1–14)	6.9 (3.5)		6.0 (0–19)	6.1 (3.5)		0.137
		Anxiety	8.0 (0–21)	8.5 (4.4)		5.0 (0–18)	5.5 (3.4)		<0.001
		Social dysfunction	9.0 (6–16)	9.4 (2.6)		7.0 (3–16)	8.1 (2.4)		0.002
		Depression	0.0 (0–12)	1.8 (3.0)		0.0 (0–6)	0.3 (0.9)		<0.001
		Sum case score	7.0 (0–21)	7.6 (5.6)		3.0 (0–24)	4.6 (4.4)		0.001
	**IES**	Intrusion	22.5 (1–40)	21.1 (10.7)	48	8.0 (0–29)	9.8 (6.6)	105	<0.001
		Avoidance	9.0 (0–30)	9.4 (7.2)		1.0 (0–26)	2.5 (4.1)		<0.001
		Arousal Likert	10.5 (0–28)	11.6 (7.8)		3.0 (0–25)	3.8 (4.3)		<0.001
	**EPDS**	Sum	10.0 (1–24)	10.5 (6.0)	47	2.0 (0–17)	3.1 (3.1)	105	<0.001
**Time 2**	**GHQ**	Sum Likert	22.0 (9–41)	23.8 (9.2)	48	16.0 (3–50)	16.7 (7.2)	105	<0.001
		Health perception	6.5 (1–16)	6.7 (3.4)		4.0 (0–17)	4.9 (3.2)		0.002
		Anxiety	7.0 (0–16)	7.2 (3.8)		4.0 (0–14)	4.1 (2.8)		<0.001
		Social dysfunction	8.0 (6–14)	8.8 (2.5)		7.0 (0–16)	7.6 (2.3)		0.004
		Depression	1.0 (0–12)	1.9 (2.9)		0.0 (0–9)	0.4 (1.1)		<0.001
		Sum case score	4.5 (0–17)	6.0 (5.3)		2.0 (0–18)	2.8 (3.7)		<0.001
	**IES**	Intrusion	14.0 (1–36)	15.8 (9.7)	48	5.0 (0–25)	6.9 (6.3)	105	<0.001
		Avoidance	5.0 (0–27)	6.6 (6.7)		0.0 (0–14)	1.3 (2.5)	104	<0.001
		Arousal Likert	5.0 (0–23)	7.3 (6.2)		2.0 (0–20)	3.0 (3.8)	104	<0.001
	**EPDS**	Sum	6.0 (0–17)	6.1 (4.6)	48	2.0 (0–15)	2.5 (3.0)	105	<0.001
**Time 3**	**GHQ**	Sum Likert	21.0 (9–41)	22.2 (9.0)	47	17 (7–49)	18.8 (7.9)	105	0.030
		Health perception	5.0 (1–14)	6.2 (3.5)	48	5.0 (0–16)	5.7 (3.6)		0.428
		Anxiety	6.0 (0–13)	6.7 (3.7)	47	4.0 (0–15)	4.9 (3.0)		0.004
		Social dysfunction	8.0 (6–17)	8.3 (2.3)	47	7.0 (2–18)	8.0 (2.4)		0.290
		Depression	0.0 (0–8)	0.8 (1.7)	48	0.0 (0–7)	0.3 (0.9)		0.010
		Sum case score	4.0 (0–17)	5.1 (4.8)	47	2.0 (0–21)	3.9 (4.4)		0.170
	**IES**	Intrusion	8.0 (1–30)	11.6 (8.8)	48	5.0 (0–31)	7.1 (6.8)	105	0.002
		Avoidance	4.0 (0–30)	5.2 (7.0)	47	0.0 (0–22)	1.4 (3.3)		<0.001
		Arousal Likert	4.0 (0–24)	6.5 (6.4)	47	2.0 (0–20)	3.5 (3.7)		0.008
	**EPDS**	Sum	9.0 (0–18)	8.4 (4.8)	48	4.0 (0–18)	5.2 (4.9)	105	<0.001
**Time 4**	**GHQ**	Sum Likert	18.0 (7–52)	21.4 (10.3)	48	19.0 (6–56)	19.6 (7.9)	105	0.507
		Health perception	5.0 (1–17)	6.2 (4.2)		5.0 (1–19)	6.2 (3.8)		0.805
		Anxiety	6.0 (0–17)	6.4 (3.9)		5.0 (0–17)	5.0 (2.9)		0.039
		Social dysfunction	8.0 (1–19)	8.4 (2.8)		8.0 (1–17)	8.3 (2.6)		0.687
		Depression	0.0 (0–10)	0.4 (1.6)		0.0 (0–5)	0.1 (0.6)		0.063
		Sum case score	3.5 (0–21)	5.0 (5.3)		3.0 (0–23)	4.5 (4.3)		0.921
	**IES**	Intrusion	7.0 (0–33)	10.5 (8.5)	48	5.0 (0–28)	8.1(7.6)	105	0.067
		Avoidance	3.0 (0–35)	5.2 (7.8)		0.0 (0–14)	1.0 (2.5)		<0.001
		Arousal Likert	3.0 (0–27)	5.7 (6.8)		3.0 (0–21)	3.3 (3.5)		0.083
	**EPDS**	Sum	4.0 (0–17)	5.2 (4.8)	48	2.5 (0–16)	2.9 (2.8)	104	0.012

**Abbreviations:** IES, Impact of Event Scale; GHQ, General Health Questionnaire; EPDS, Edinburgh Postnatal Depression Scale; SD, standard deviation; Time 1, at recruitment; Time 2, 2–3 weeks after recruitment; Time 3, at 30 gestational weeks; Time 4, at 36 gestational weeks.

* Independent sample Mann–Whitney U Test.

**Fig 1:**
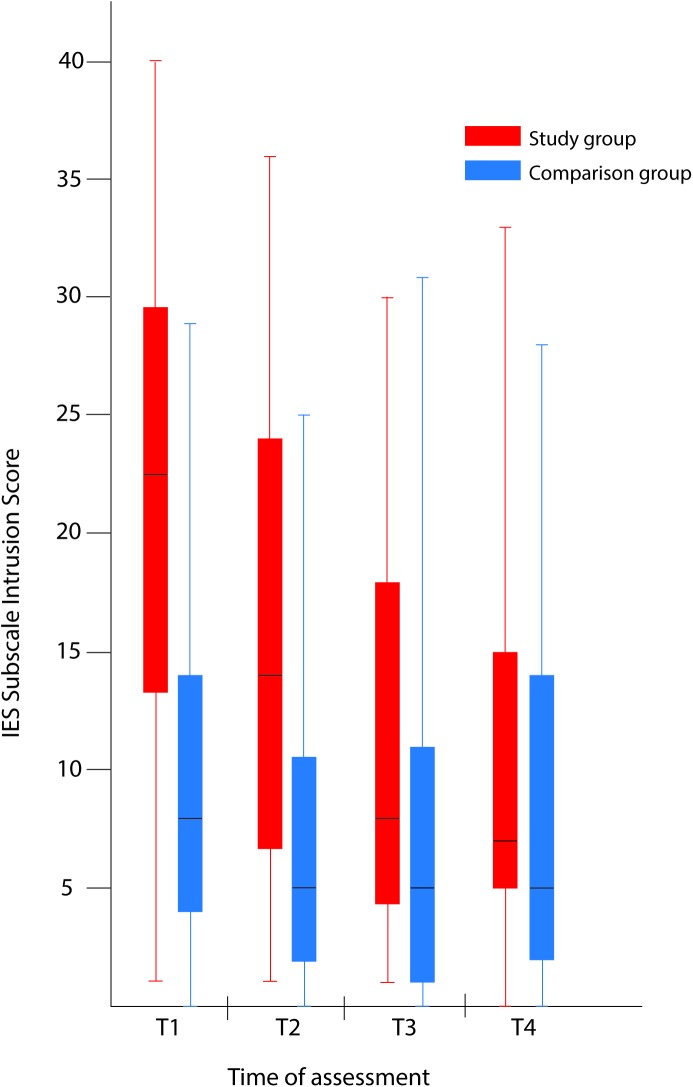
Comparison of maternal psychological distress during pregnancy with and without a fetal anomaly. The figure presents psychological distress in women in the study group (with fetal anomaly) and comparison group (normal ultrasound findings) at the four assessments, as measured by the Intrusion subscale of the Impact of Event Scale (IES). Box-and-whiskers plots show 50% of cases (25–75 percentiles) in the rectangle, each of the whiskers represents the smallest and largest values. The line within the rectangle represents the median value.

The psychometric IES scores were significantly higher in the study group than in the comparison group at T1, T2, and T3 (Table 2 and Fig 1). At T4, the differences between the groups were significant for the IES avoidance score only.

In the study group, there were 23(48%), 13(27%), eight (17%) and four (8%) women with an IES Intrusion score ≥20 and GHQ case score ≥6 at T1, T2, T3, and T4, respectively; three women had a score above these levels at all four assessments. In the comparison group, there were five (5%), two (2%), two (2%), and five (5%) women with an IES intrusion score ≥20 and GHQ case score ≥6 at T1, T2, T3, and T4; one (1%) woman had high scores at T2, T3, and T4, but none had high scores at all assessments.

**Fig 2:**
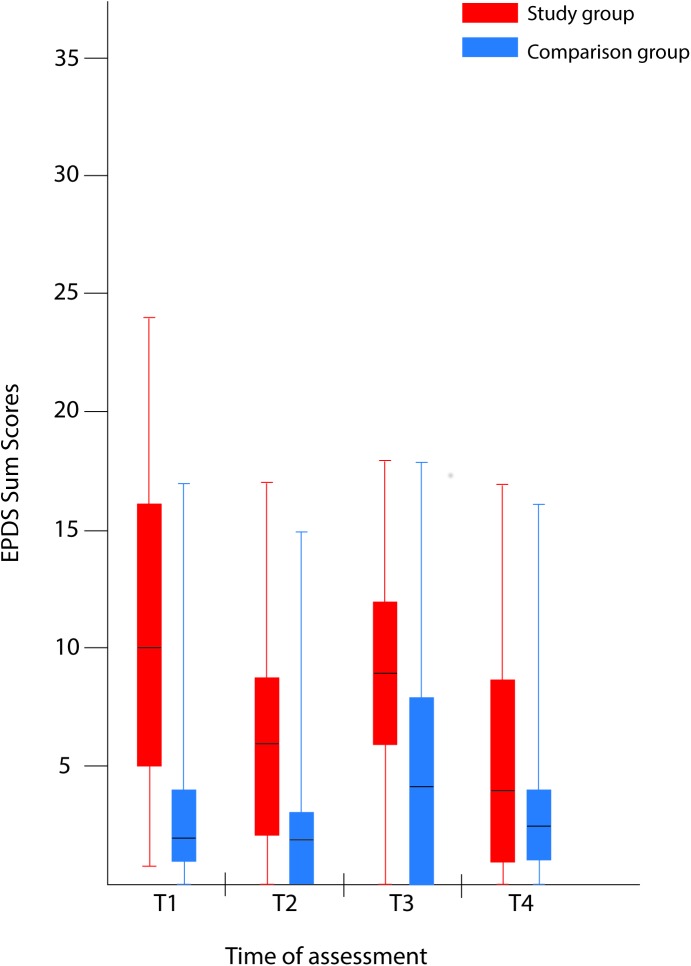
Comparison of maternal psychological distress during pregnancy with and without a fetal anomaly. The figure presents psychological distress in women in the study group (with fetal anomaly) and comparison group (normal ultrasound findings) at the four assessments, as measured by the Edinburgh Postnatal Depression Scale (EPDS). Box-and-whiskers plots show 50% of cases (25–75 percentiles) in the rectangle, each of the whiskers represents the smallest and largest values. The line within the rectangle represents the median value.

The EPDS total score was significantly higher in the study group, at all of the assessments (Table 2 and Fig 2).

At T1, the study group showed high psychometric scores, with median GHQ case score 7, IES Intrusion subscale 22.5, and EPDS total score 10. The scores were lower at T2 and were even lower at T3, with minor changes seen from T3 to T4. The EPDS score increased slightly from T2 to T3 but decreased at T4 (Table 2, Figs 1 and 2). In the study group, clinically important intrusion was found in 30 of 48 women at T1.

The comparison group had median psychometric scores within the normal range on all the psychometric questionnaires (i.e., the GHQ case score, subscales of the IES, and the EPDS total score), with the exception of the IES Intrusion subscale at T1; however, 36 of the 105 women in the comparison group had case scores above the cut-off level for clinical relevance (on the GHQ) at T4.

In the study group, the fetal diagnosis/prognosis changed between T1 and T2 in 20 women. Two had a worsening and 18 an improvement in the prognosis. The women with improved fetal prognosis all received a normal test result from karyotyping before T2. The two women with a worse fetal prognosis received an abnormal karyotype test result (trisomy 21) and had high scores at all four assessments on all nine psychometric outcomes. Both were in the older age group (i.e. range 34–43 years), and one had a previous child with a congenital anomaly. Initially they both had the fetal anomaly classified as ‘severity not classified; anomaly awaiting clarification’. The group of women with an improved fetal diagnosis and prognosis were significantly older and had significantly higher education than the women with a stable fetal diagnosis/prognosis; in this group, a high number had the fetal anomaly classified as ‘severity not classified; anomaly awaiting clarification’ until the result from the chromosome test was available. There was no significant difference in psychometric stress scores between women with improved diagnosis and those with stable diagnosis at any of the assessments, with the exception of a difference in the GHQ social dysfunction (i.e., emotional problems largely experienced in social situations) score at T2.

The number of women with a score ≥3 on any of the IES items or ≥1 on any of the EPDS items at all four assessments differed between the groups, with the higher number found in the study group. These high scores all derived from items related to depression (Table 3).

**Table 3:** Number of women with a score ≥ 3 on any of the IES items and ≥ 1 at any of the EPDS items at all four assessments. High score were considered ≥ 3 for the IES and ≥ 1 for EPDS.

Question-naire	Item	Question/wording	Study group N = 48 (N, %)	Comp-arison group N = 105 (N, %)	P-value*
**IES**	**# 1**	Any reminder brought back the feelings about it	14 (50)	24 (23)	0.524
	**# 2**	I had trouble staying asleep	8 (17)	13 (12)	0.644
	**# 3**	Other things kept making me think about it	9 (19)	4 (4)	0.006
	**# 4**	I felt irritable and angry	11 (23)	8 (8)	0.016
	**# 6**	I thought about it when I didn’t mean to	7 (15)	1 (1)	0.002
	**# 20**	I had trouble concentrating	9 (19)	0	<0.001
**EPDS**	**# 3**	I have blamed myself unnecessarily when things went wrong	11 (23)	5 (5)	0.002
	**# 4**	I have been anxious or worried for no good reason	11 (23)	10 (10)	0.048
	**# 6**	Things have been getting on top of me	24 (50)	14 (13)	<0.001
	**# 7**	I have been so unhappy that I have had difficulty sleeping	10 (21)	0	<0.001
	**# 8**	I have felt sad and miserable	18 (38)	2 (2)	<0.001
	**# 9**	I have been so unhappy that I have been crying	11 (23)	0	<0.001

**Abbreviations:** IES Impact of Event Scale, EPDS Edinburgh Postnatal Depression Scale.

* Chi-squared test.

Univariate ANOVA with one independent variable (i.e., fetal diagnostic and prognostic classification, gestational age at recruitment, change in diagnosis/prognosis, maternal age, parity, or education) in each analysis, for each IES outcome variable at T1 did not disclose any major significant trends, except some border significant values. Adjusted ANOVA with variables (i.e. fetal diagnostic and prognostic classification, gestational age at recruitment, maternal age, and education) gave border significance for fetal diagnostic and prognostic classification, with highest stress in women with the more severe fetal prognostic classification. See S1 Table.

Concerning suicidal ideation there was a significant difference between the groups (Chi-square analysis, p = 0.006). In the study group, seven (15%) women had GHQ scores indicating suicidal ideation at 16 items (GHQ 24, 25, 27, 28 or EPDS 10): eight at T1 (16%), one at T2 (2%), four (8%) at T3, and three at T4 (6%). In the comparison group, two (2%) women had suicidal ideation at T1. Three women in the study group required intervention (appointment with a psychiatrist or with a municipal health care worker). None committed suicide. Performing Chi-square analyses there were no significant differences in the demographic variables presented in Table 1 between those with suicidal ideation versus the others (p-values 0.979–0.100). See S2 Table for more information.

## Discussion

### Main findings

Our main finding was that maternal psychological distress after detection of a fetal anomaly by ultrasound declined during the pregnancy, from an initial high level to almost normal level but with persistent symptoms of depression towards the end of pregnancy. There was no significant difference between the psychometric scores of women who had an improved fetal prognosis and those with a stable prognosis, with the exception of a small, clinically unimportant difference in GHQ score for social dysfunction (emotional problems largely experienced in social situations) at T2.

The decline in psychometric stress scores in the study group may reflect a general phenomenon in response to stressful life events. [18, 19] This hypothesis is supported by a study by Titapant and Chuenwattana [3] who showed a general decrease in anxiety levels over pregnancy, with an increase at 37 weeks gestation. To our knowledge, this is the only longitudinal study of distress in pregnancies with a prenatally diagnosed fetal anomaly. In a Norwegian population-based study by Nes et al., a reduction of maternal psychological distress was found, from week 18 to week 30, in pregnancies with a fetal anomaly—notably the authors did not specify whether the women were aware of the fetal anomaly before the birth. [4]

Our comparison group showed small variations in psychometric scores from T1 to T4. This is similar to the finding of stable psychological stress levels in pregnancies without fetal anomaly, reported by Nes et al. [4] Contrary to the results in our comparison group, a British study found an increase in depression and anxiety from gestational week 18 to 32, [20] whereas an Australian study described a decline in stress and anxiety scores from week 16 to week 28, followed by an increase until week 32. [21] Moreover, Liou et al. [22] reported a decrease in stress and increase in anxiety levels during pregnancy, and also found a transient elevation of depressive symptoms from week 25 to 29. These studies all investigated normal pregnancies.

The women in the study group with high scores on the IES and EPDS (Table 3) predominantly scored high on the questions related to depression. Previous studies have shown that factors associated with peripartum depression include traumatic or stressful life events in the previous year, [23, 24] which apply to our study group.

Structural anomalies appearing on an ultrasound examination may not give sufficient information to provide an exact diagnosis and prognosis, e.g., in the case of a syndrome. A chromosome test may be indicated, as was the case in the groups with improved or worse fetal diagnosis following the chromosomal analysis. These subgroups were characterised by older women with a higher educational level than in the subgroup with a stable diagnosis and were apparently, a specific subgroup with an increased risk of fetal aneuploidy due to maternal age. The two women with a worse fetal prognosis following chromosomal analysis had high and only minor changes in psychometric distress scores during pregnancy. Notably, one was single and had a previous child with special needs. In a retrospective study including 40 women, Horsch et al. [25] concluded that such factors influence maternal coping and adjustment following a diagnosis of fetal anomaly.

A lower educational level among the women in our study group could increase their level of distress. The study group was referred to the fetal medical unit from the whole country. The women in the comparison group were all scheduled to deliver at the hospital where they also had their routine ultrasound examination. The women scheduled to deliver at the hospital are possibly somewhat older and from a relatively higher social class which corresponds to a higher educational level than the average Norwegian fertile female population. The average age at birth in Norway is 30.6 years (www.fih.no/hn/helseregistre-og-registre/mfr), while the average age in the study group was 30.0 years and in the comparison group 31.7 years. This could hardly bias the results. The prevalence of distress and mental disorders has been found to be negatively related to educational level; [26] however, in our study, ANOVA did only reveal an association between education level and stress scores (GHQ, IES and EPDS) at T1 for IES arousal (see S1 Table). Thus, the comparatively lower educational level in the study group cannot explain our findings.

We did not observe an increase in stress level at the last assessment before delivery, in any of the subgroups, contrary to the findings of the study on pregnancy with non-lethal malformation, from Thailand. [3] The Thai study used the Spielberger State Anxiety Inventory, which focuses temporary and situational anxiety, to assess anxiety during pregnancy. The present study used three different questionnaires, one covering two weeks (GHQ), the two others (IES and EPDS) the previous week to the assessment. This difference in questionnaire focus may explain why we did not find an elevation in anxiety or stress scores at the last assessment before delivery.

For most women, the transition to parenthood is part of the normal ‘burden’ of living, whereas awareness of a fetal anomaly interferes with quality of life and may increase the risk of developing psychopathological symptoms. [27] The way a person copes with such a situation will depend on variables related to personal vulnerability and the ability to handle life events, e.g., way of thinking, attitudes, and cognitive style. None of the variables we tested could explain the stress level in these women. Other researchers have found attitude and support from family and friends to be important. [14] After a crisis, the early adaption to the new situation (fetal anomaly) involves a reorientation phase partially depending on the factors above, e.g., ability to handle life events and support. [28] In the study group, we observed a decline in psychological stress levels throughout pregnancy, which accords with our clinical impression. At the four assessments, the women in the study group met the same health professionals who knew their medical situation and were supportive. [29] By exploring the women’s concerns and needs related to the fetal diagnosis, the health providers helped the women implement coping strategies and mitigate the negative effects of uncertainty. [30] Ultrasound diagnosis of anomalies most likely has a ‘psychological cost’ that possibly can be reduced by knowledge-based care. [31] Hunfeld et al. [32] suggested that perinatal team counselling that provides clear and consistent information concerning the anomaly, including the prognosis, reduces stress and uncertainty. Our data did not include distress scores related to the delivery of information.

### Strengths and limitations

The strengths of this study include a prospective longitudinal design, use of repeated assessments (four) based on three standardised psychometric methods, and inclusion of a comparison group. We followed the participating women from the diagnostic ultrasound examination to 36 weeks; thus, we were able to follow changes in psychometric scores as well as in fetal diagnosis and prognosis. The study group has to be considered as a selected group because they have either decided to continue their pregnancy or the fetal anomaly did not give the legal option to terminate pregnancy.

In a previous study, [2] we found that gestational age had an impact on the level of psychological stress in pregnant women with a fetal anomaly. The present study group varied in gestational age at recruitment, which hindered the use of regular time series analysis.

The high scores on items identifying suicidal ideation led to supervision and thorough evaluation, with a same-day appointment with a professional. We cannot rule out the possibility that intervention for those reporting suicidal ideation contributed to somewhat lower distress scores in the total group.

Convenience sampling may have a potential for selection bias because included women are not a random sample of the total population. We aimed to minimize this problem by including women from a fixed date and only stopping inclusion when the workload made inclusion impossible.

A wide variation of gestational age in the study group at inclusion is a challenge (some of the included women could not attend all four assessments due to late inclusion), but reflects our patient population in fetal medicine. Detection of fetal anomalies occurs throughout pregnancy.

The sample size was small, and we have considered this. Taking into account an assessment of the relation between the expected differences between the groups and an a priori border for Type I error (5%) and Type II error (85%), 40 women in each group was adequate. [2] We have been cautious not to draw strong conclusions in our sub-analyses because the groups will be smaller than the calculated 40 women.

The difference in educational level between the groups can possible skew the results, since level of education may have an influence on psychological stress [33, 34], but performing unadjusted and adjusted ANOVA educational level did not influence the stress level (GHQ, IES and EPDS), except in adjusted analysis for IES arousal. See S1 Table.

### Conclusion

Pregnant women with fetal anomalies identified in the second trimester initially had high psychological stress levels; however, during pregnancy, their level of stress decreased compared to women with normal ultrasound examinations. In women with a normal pregnancy, psychological stress levels were relatively stable and remained low through the last half of gestation, although large individual differences were found. Stress levels did not increase toward the end of pregnancy (36 weeks gestation) in either of the groups.

## Supporting information

S1 Table Unadjusted and adjusted mean values (95% CI) of IES subscales, GHQ and EPDS as dependent variable in the study group (n = 48) using ANOVA. (DOCX)

S2 Table Characteristics for women in study group with and without suicidal ideation (T1). (DOCX)
