# Supplementary material for: Maternal psychological responses during pregnancy after ultrasonographic detection of structural fetal anomalies: A prospective longitudinal observational study
Source: PLoS One. 2017 Mar 28;12(3):e0174412. doi: 10.1371/journal.pone.0174412 (PMC5369773; doi:10.1371/journal.pone.0174412)
Supplement: S1 Table — (DOCX) [file pone.0174412.s001.docx]

| Independent  variable | | Dependent variable T1 | | | | | | | |
| --- | --- | --- | --- | --- | --- | --- | --- | --- | --- |
|  | | IES Intrusion (n=48) | | | | IES Avoidance (n=48) | | | |
|  |  | Unadjusted | | Adjusted | | Unadjusted | | Adjusted | |
|  |  | Mean (CI) | P-value | Mean (CI) | P-value | Mean (CI) | P-value | Mean (CI) | P-value |
| Fetal diagnostic and prognostic classification* | 2  3  4  5 | 20.0 (11.3-28.7)  17.9 (10.8-25.0)  18.8 (13.3-24.3)  25.1 (20.0-30.1) | 0.260 | 17.8 (7.8-27.8)  17.0 (8.7-25.3)  18.1 (11.0-25.3)  27.8 (21.7-33.9) | 0.075 | 7.2 (1.5-12.8)  10.9 (6.3-15.5)  5.9 (2.4-9.5)  12.4 (9.1-15.7) | 0.053 | 7.9 (1.6-14.3)  13.0 (7.7-18.3)  7.3 (2.8-11.8)  13.8 (10.0-17.7) | 0.067 |
| Maternal age (years) | 19-28  29-33  34-43 | 22.4 (17.1-27.7)  21.1 (16.2-26.0)  19.2 (12.6-25.8) | 0.744 | 20.4 (13.9-26.9)  22.6 (16.5-28.7)  17.6 (9.4-25.7) | 0.491 | 9.6 (6.2-13.1)  7.2 (4.0-10.3)  13.3 (9.0-17.5) | 0.076 | 10.5 (6.4-14.6)  8.0 (4.1-11.9)  13.1 (7.9-18.2) | 0.169 |
| Previous children | No  Yes | 19.8 (15.0-24.6)  22.1 (18.0-26.2) | 0.473 |  |  | 9.9 (6.6-13.2)  9.1 (6.3-11.9) | 0.713 |  |  |
| Education | >Junior college  ≥Junior college | 21.7 (17.2-26.3)  20.6 (16.2-24.9) | 0.706 | 22.2 (16.9-27.5)  18.1 (11.3-25.0) | 0.267 | 9.7 (6.7-12.8)  9.2 (6.2-12.1) | 0.785 | 11.4 (8.1-14.8)  9.6 (5.3-13.9) | 0.420 |
| Gestational age at T1 (weeks) | >18  18-22  <22->27 | 18.9 (12.4-25.4)  21.1 (17.3-24.8)  27.5 (16.7-38.3) | 0.395 | 15.9 (8.8-23.0)  19.9 (15.9-24.0)  24.7 (13.1-36.3) | 0.379 | 9.1 (4.7-13.5)  9.0 (6.4-11.5)  14.3 (6.9-21.6) | 0.390 | 8.0 (3.5-12.5)  9.6 (7.0-12.1)  14.0 (6.6-21.3) | 0.384 |
| Time from suspicion of fetal anomaly to examination at the referral center (days) | ≤2  3-4  ≥5 | 20.7 (17.1-24.3)  18.8 (9.1-28.5)  25.0 (16.8-33.2) | 0.553 |  |  | 10.5 (8.2-12.9)  3.6 (-2.8-10.0)  8.0 (2.6-13.4) | 0.113 |  |  |
| Change in diagnosis/prognosis | Improvement  Stable  Worsening | 21.6 (16.4-26.8)  20.7 (16.5-24.8)  23.0 (7.5-38.5) | 0.931 |  |  | 9.3 (5.9-12.6)  8.8 (6.1-11.5)  20.0 (10.0-30.0) | 0.105 |  |  |

**S1 Table. Unadjusted and adjusted mean values (95 % CI) of IES subscales, GHQ and EPDS as dependent variable in the study group (n=48) using ANOVA**

| Independent  variable | | Dependent variable T1 | | | |
| --- | --- | --- | --- | --- | --- |
|  | | IES arousal (n=48) | | | |
|  |  | Unadjusted | | Adjusted | |
|  |  | Mean (CI) | P-value | Mean (CI) | P-value |
| Fetal diagnostic and prognostic classification* | 2  3  4  5 | 6.5 (0.1-12.9)  12.4 (7.3-17.6)  10.5 (6.4-14.5)  13.8 10.1-17.4) | 0.230 | 3.9 (-3.1-10.9)  12.7 (6.9-18.5)  9.2 (4.2-14.1)  15.6 (11.4-19.8) | 0.025 |
| Maternal age (years) | 19-28  29-33  34-43 | 12.9 (9.0-16.8)  10.2 (6.6-13.8)  12.1 (7.3-16.9) | 0.577 | 11.6 (7.0-16.1)  9.6 (5.4-13.9)  9.8 (4.1-15.5) | 0.772 |
| Previous children | No  Yes | 14.1 (10.7-17.5)  9.8 (6.9-12.7) | 0.059 |  |  |
| Education | >Junior college  ≥Junior college | 13.3 (10.0-16.5)  10.0 (6.9-13.2) | 0.157 | 12.9 (9.3-16.6)  7.7 (3.0-12.5) | 0.045 |
| Gestational age at T1 (weeks) | >18  18-22  <22->27 | 9.5 (4.8-14.3)  11.7 (9.0-14.5)  16.0 (8.1-23.9) | 0.372 | 7.1 (2.1-12.1)  11.3 (8.4-14.1)  12.7 (4.5-20.8) | 0.265 |
| Time from suspicion of fetal anomaly to examination at the referral center (days) | ≤2  3-4  ≥5 | 11.9 (9.3-14.5)  6.4 (-0.6-13.4)  13.6 (7.6-19.5) | 0.264 |  |  |
| Change in diagnosis/prognosis | Improvement  Stable  Worsening | 11.9 (8.1-15.7)  11.5 (8.5 14.6)  9.5 (-1.9-20.9) | 0.922 |  |  |

| Independent  variable | | Dependent variable T1 | | | | | | | |
| --- | --- | --- | --- | --- | --- | --- | --- | --- | --- |
|  | | GHQ total sum Likert score (n=48) | | | | EPDS sum (n=48) | | | |
|  |  | Unadjusted | | Adjusted | | Unadjusted | | Adjusted | |
|  |  | Mean (CI) | P-value | Mean (CI) | P-value | Mean (CI) | P-value | Mean (CI) | P-value |
| Fetal diagnostic and prognostic classification* | 2  3  4  5 | 21.3 (12.4-30.2)  27.0 (19.7-34.3)  25.8 (20.2-31.4)  28.7 (23.6-33.9) | 0.537 | 20.3 (10.0-30.6)  26.4 (17.9-35.0)  26.0 (18.6-33.3)  31.9 (25.7-38.2) | 0.228 | 9.7 (4.8-14.6)  11.8 (7.8-15.8)  8.3 (5.2-11.4)  12.2 (9.3-15.1) | 0.279 | 8.3 (2.8-13.8)  11.3 (6.7-16.0)  7.9 (3.9-11.8)  13.8 (10.4-17.2) | 0.083 |
| Maternal age (years) | 19-28  29-33  34-43 | 26.4 (21.0-31.7)  27.1 (22.1-32.0)  26.0 (19.3-32.7) | 0.963 | 25.3 (18.6-32.0)  28.1 (21.8-34.4)  25.2 (16.7-33.6) | 0.716 | 10.6 (7.6-13.7)  10.3 (7.5-13.2)  10.7 (7.0-14.5) | 0.980 | 10.0 (6.4-13.6)  10.9 (7.4-14.3)  10.1 (5.6-14.7) | 0.916 |
| Previous children | No  Yes | 29.1 (24.3-33.9)  24.8 (20.7-28.8) | 0.169 |  |  | 11.8 (9.0-14.5)  9.6 (7.3-12.0) | 0.237 |  |  |
| Education | >Junior college  ≥Junior college | 28.0 (23.5-32.5)  25.2 (20.9-29.6) | 0.379 | 28.6 (23.1-34.0)  23.8 (16.8-30.8) | 0.209 | 11.3 (8.8-13.8)  9.8 (7.3-12.3) | 0.396 | 11.8 (8.9-14.8)  8.8 (5.1-12.6) | 0.136 |
| Gestational age at T1 (weeks) | >18  18-22  <22->27 | 24.9 (18.4-31.4)  26.2 (22.5-30.0)  33.8 (23.0-44.5) | 0.360 | 21.6 (14.3-29.0)  25.3 (21.1-29.5)  31.6 (19.6-43.6) | 0.348 | 8.2 (4.6-11.8)  10.8 (8.7-12.9)  14.8 (8.8-20.7) | 0.158** | 6.8 (2.8-10.7)  10.8 (8.6-13.1)  13.4 (6.9-19.8) | 0.111 |
| Time from suspicion of fetal anomaly to examination at the referral center (days) | ≤2  3-4  ≥5 | 25.9 (22.2-29.5)  26.6 (16.8-36.4)  30.0 (21.7-38.3) | 0.660 |  |  | 10.6 (8.6-12.6)  7.3 (1.2-13.3)  12.1 (7.5-16.8) | 0.439 |  |  |
| Change in diagnosis/prognosis | Improvement  Stable  Worsening | 27.1 (21.9-32.2)  26.5 (22.3-30.7)  23.0 (7.4-38.6) | 0.883 |  |  | 11.6 (8.7-14.6)  9.9 (7.5-12.2)  10.5 (1.8-19.2) | 0.637** |  |  |

**Abbreviations:** IES, Impact of Event Scale - 22 R; GHQ, General Health Questionnaire - 28; EPDS, Edinburgh Postnatal Depression Scale. *See text. None classified as 1. ** Levine’s Test of Equality of Error Variances > 0.05
