# Supplementary material for: Maternal psychological responses during pregnancy after ultrasonographic detection of structural fetal anomalies: A prospective longitudinal observational study
Source: PLoS One. 2017 Mar 28;12(3):e0174412. doi: 10.1371/journal.pone.0174412 (PMC5369773; doi:10.1371/journal.pone.0174412)
Supplement: S2 Table — (DOCX) [file pone.0174412.s002.docx]

**S2 Table Characteristics for women in study group with and without suicidal ideation (T1)**

|  | **Women with all four assessments/visits** | | |
| --- | --- | --- | --- |
|  | **Suicidal ideation (n=7)**  **N (%)** | **Without suicidal ideation (n=41)**  **N (%)** | **P-value*** |
| **Age**  19-28 years  29-33 years  34-43 years | 2  3  2 | 15  17  9 | 0.893 |
| **Education**  < junior college  ≥ junior college  Missing data | 3  4 | 20  21 | 0.100 |
| **Previous children**  no previous children  previous children | 4  3 | 16  25 | 0.429 |
| **Married or cohabiting**  Married/cohabitating  Not cohabiting | 7  0 | 39  2 | 0.100 |
| **Gestational age at first assessment**  <18 weeks  18-22 weeks  >22 – <27 weeks | 1  6  0 | 10  27  4 | 0.524 |
| **Time from suspicion of fetal anomaly to examination at the referral center**  **≤ 2 days**  **3-4 days**  **≥ 5 days** | 5  1  1 | 31  4  6 | 0.936 |
| **Change in diagnosis/prognosis from T1 to T2**  Improvement  Stable  Worsening | 13  26  2 | 5  2  0 | 0.129 |
| **Classification of severity (see text)**  1  2  3  4  5 | 0  1  1  2  3 | 0  5  8  13  15 | 0.979 |

***** Chi-Square (Pearson Chi-Square, Continuity Correction, or Fisher’s Exact Test as appropriate)
